# Unraveling the Roles of UBE3A in Neurodevelopment and Neurodegeneration

**DOI:** 10.3390/ijms26052304

**Published:** 2025-03-05

**Authors:** Xin Yang, Yu-Wen Alvin Huang

**Affiliations:** 1 The Department of Molecular Biology, Cell Biology & Biochemistry, Brown University, Providence, RI 02903, USA; 2 Carney Institute for Brain Science, Brown University, Providence, RI 02903, USA; 3 Center for Translational Neuroscience in Brown Institute for Translational Sciences, Brown University, Providence, RI 02903, USA

**Keywords:** UBE3A, autism spectrum, Angelman syndrome, brain disorders, neuron–glia interactions, neurodegeneration

## Abstract

The ubiquitin-protein ligase E3A (UBE3A, aka E6-AP), an E3 ligase belonging to the HECT family, plays crucial roles in the stability of various proteins through the proteasomal degradation system. Abnormal UBE3A activity is essential for the initiation and progression of several cancers. A gain of function and an overdosage of maternal UBE3A is associated with an increased risk of autism spectrum disorders. Conversely, a loss of function due to mutations, deletions, paternal duplications, or imprinting defects in neurons leads to Angelman syndrome. Emerging evidence suggests that abnormal UBE3A activity may also contribute to the development of various brain disorders, including schizophrenia, Huntington’s disease, Parkinson’s disease, and Alzheimer’s disease, making UBE3A a protein of significant interest. However, research on UBE3A’s functions in the brain has primarily focused on neurons due to the imprinting of UBE3A in mature neuronal cells, while being obscured in glia. This review outlines the expression of UBE3A in neurons and glial cells based on published studies, highlights newly identified patterns of UBE3A, such as its secretion, and emphasizes the involvement of UBE3A in neurodegenerative diseases. Furthermore, we summarize glial UBE3A and propose a model of bi-directional interactions between the neurons and glia mediated by UBE3A that underlies brain functions. Insights gained from this research could provide new avenues for therapeutic interventions targeting various brain disorders.

## 1. Introduction

The ubiquitin-protein ligase E3A, UBE3A (aka. E6-AP), is an E3 ligase of the HECT family. It is essential for the stability of various proteins through the proteasomal degradation system (readers interested in the proteasomal degradation system can refer to recent reviews [1,2]). It interacts with the E6 protein expressed by infected human papillomavirus (HPV) underlying the initiation and progression of several cancers [3,4]. In normal situations, especially during brain development, UBE3A expression must be tightly controlled. Located on the chromosome region 15q11-13, UBE3A is maternally expressed in mature neurons while being biallelically expressed in other cells [5,6,7,8]. Therefore, it has become more attractive to understand UBE3A in neurons, specifically the maternal UBE3A expression, paternal imprinting mechanisms, and cellular and molecular functions of neuronal UBE3A. It has been well studied that the gain of function and overdosage, especially duplication, of maternal UBE3A, causes penetrance of the autism spectrum (Dup15q specifically). Loss of function due to mutations, deletions, paternal duplications, or imprinting defects in neurons leads to another neurodevelopmental disorder: Angelman syndrome [OMIM #105830] [9]. Potential pathological mutations of UBE3A could be found in Uniprot (https://www.uniprot.org/uniprotkb/Q05086/variant-viewer, accessed on 31 August 2024) and ClinVar at NCBI (https://www.ncbi.nlm.nih.gov/clinvar?term=601623[MIM], accessed on 31 August 2024). Currently, there are no available therapies for Dup15q syndrome and Angelman syndrome, and there is an urgent need to develop effective therapeutics. Interestingly, Dup15q syndrome and Angelman syndrome often share common symptoms and deficits, including seizures, sleep problems, and cognitive impairments. These may indicate that they involve the same or similar neuronal circuits, signaling pathways, or molecular mechanisms. Since UBE3A is imprinted in mature mammalian CNS neurons, past research on UBE3A has been largely restricted to neurons. Recent breakthroughs have provided significant insight into UBE3A functions and potential interventions, such as the findings on the effects of the UBE3A antisense oligonucleotide(Ube3a-ASO) [9,10], paternal UBE3A unsilencer [11,12,13], and UBE3A activators [14,15]. However, the exact mechanisms whereby alternations of UBE3A expression and protein structures result in widespread brain anomalies remain largely unclear. 

Strikingly, growing evidence supporting aberrant UBE3A activity [16] would contribute to the manifestations of an array of additional neurological disorders including schizophrenia [17], Huntington’s [18,19], Parkinson’s [20], and Alzheimer’s diseases [21,22,23], making UBE3A a protein of high research and clinical interest. Therefore, thoroughly exploring UBE3A functions is crucial for developing therapeutic interventions for neuropsychiatric diseases. It is becoming increasingly clear that UBE3A plays a significant role in various signaling pathways related to cognitive functions. Additionally, recent findings highlight the important contributions of glial cells to what were previously considered neuronal defects in neuropsychiatric disorders, prompting us to re-evaluate our understanding of UBE3A-related brain disorders. Activating paternal UBE3A for treating Angelman syndrome and manipulating UBE3A activity in conditions like Dup15q and neurodegenerative diseases are promising and worthwhile. However, many neurodevelopmental disorders, such as Angelman syndrome, have a critical window [24,25] that may limit the effectiveness of therapeutics like UBE3A reinstatement in adults, where neuronal circuits are already established and compromised [9,10,11,12,13,24,25,26,27]. Therefore, conducting foundational studies on UBE3A biology during neurodevelopment is crucial to enhance our prospects of discovering a cure for patients. This review discusses UBE3A expression in neurons and glial cells and highlights the newly identified properties of UBE3A, including its secretion and the involvement of UBE3A in neurodegenerative diseases such as Alzheimer’s disease (AD) and Huntington’s disease (HD). It emphasizes the largely unexplored roles of UBE3A in the brain glial cells and proposes potential future research directions. Insights gained from studying neuron–glia interactions involving UBE3A may provide new opportunities for therapeutic interventions in various brain disorders.

## 2. UBE3A Expression

**Figure 1:**
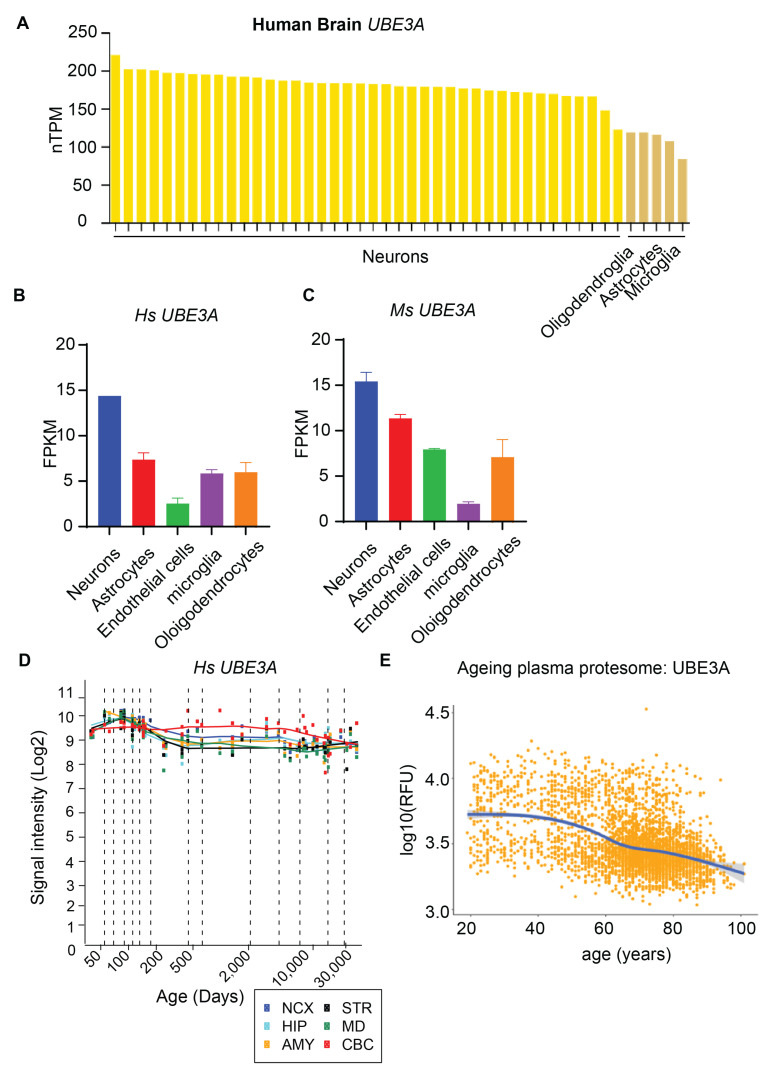
UBE3A expression in brain cells and along with human aging. (**A**) Summarized neuronal and glial UBE3A RNA expression from the human brain shown in transcripts per million (nTPM). Data were extracted from Protein Atlas https://www.proteinatlas.org/ENSG00000114062-UBE3A/single+cell+type, accessed on 31 August 2024. (**B**,**C**) Graphed comparisons of UBE3A expression (fragments per kilobase of transcript per million mapped reads, FPKM) from neurons and glia from human (**B**) and rodent brains (**C**). Data were collected [33,34] from https://brainrnaseq.org/ Zhang et al., 2014 and 2016. (**D**) Dynamic *UBE3A* expression throughout entire development and adulthood in the cerebellar cortex (CBC), mediodorsal nucleus of the thalamus (MD), striatum (STR), amygdala (AMY), hippocampus (HIP), and 11 areas of the neocortex (NCX). Obtained from the human brain transcriptome https://hbatlas.org/pages/hbtd, accessed on 16 Feb 2025. (**E**) The plot shows the detection of the UBE3A protein in plasma samples across different human age groups. (**E**) was obtained from https://twc-stanford.shinyapps.io/aging_plasma_proteome_v2/, accessed on 31 August 2024.

**Figure 2:**
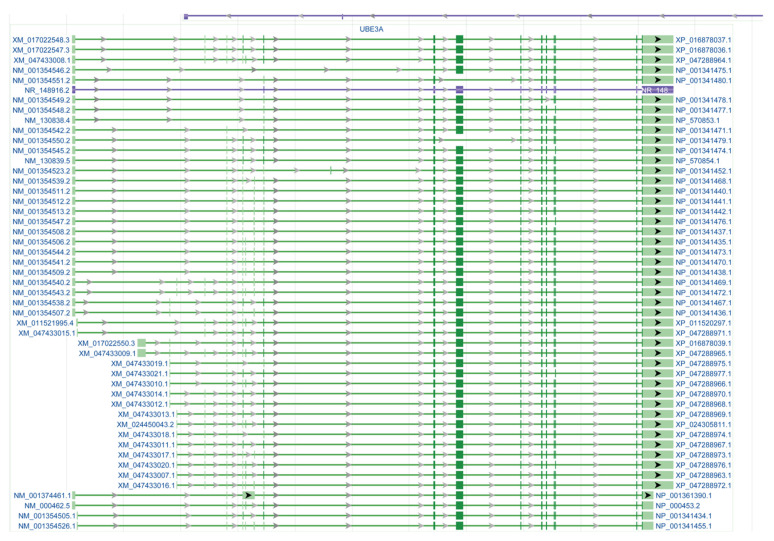
Potential UBE3A transcripts.

UBE3A is ubiquitously expressed in the brain [28]. From the available RNA sequencing database (https://brainrnaseq.org/; https://www.proteinatlas.org/ENSG00000114062-UBE3A/single+cell+type, accessed on 31 August 2024), it is evident that UBEEA is highly expressed in neurons (Figure 1A–C), which was confirmed by the immunofluorescent staining of human [29], monkey [29], and rodent [29] brain samples. However, different glia (astrocytes, oligodendrocytes, and microglia) also expressed 10–50% of UBE3A mRNA (Figure 1A–C). The aging plasma proteome database (https://twc-stanford.shinyapps.io/aging_plasma_proteome_v2/, accessed on 31 August 2024) indicated that UBE3A expression significantly decreases with aging (Figure 1D,E). It was confirmed in aging mice [19], cats, and monkeys, and human samples [30]. The decline of UBE3A with aging highlights its regulation of protein ubiquitination and degradation and potential involvement in aging-related neurodegeneration (discussed later). However, the roles of UBE3A isoforms in neurons and glial cells are still not fully understood. Traditionally, three isoforms of UBE3A proteins have been validated, with their expression being developmentally regulated. However, it has been suggested that approximately 50 UBE3A mRNA transcripts are generated through alternative splicing (Figure 2). It remains largely unknown which transcripts are expressed and when they are translated in the neurons and glial cells separately. Understanding this variability could improve our knowledge of UBE3A and aid in developing therapeutics. For instance, the specific deletion of UBE3A in the nucleus [31] has been shown to replicate the characteristics of traditional UBE3A null animals, reflecting the seizure and learning deficits observed in patients with Angelman syndrome. It may suggest that isoform and its transcriptional regulation are more critical for controlling seizures. The non-coding sequence of one UBE3A transcript regulates neuronal spine morphology and maturation through microRNA [32], implying an early-stage regulation of neuronal morphology and probably circuit formation. Therefore, UBE3A expression is developmental stage-, coding-, and non-coding-dependent, which may further challenge gene therapy in adult patients. Currently, there is no clear conclusion about which isoform(s) of UBE3A is un-silenced through the small molecules [11,13], ASO [9,10], and CRISPR techniques [27]. Developing a simple mechanism of UBE3A-dependent brain development is far more complicated. 

Around 50 transcript candidates of ubiquitin-protein ligase E3A [Homo sapiens (human)] were proposed and graphed from NCBI within the chromosome 15q11.2, assembly GRCh38.p14 at location NC_000015.10. https://www.ncbi.nlm.nih.gov/gene?Db=gene&Cmd=DetailsSearch&Term=7337, accessed on 31 August 2024.

## 3. The Activity-Dependent UBE3A Expression in Neurons

**Figure 3:**
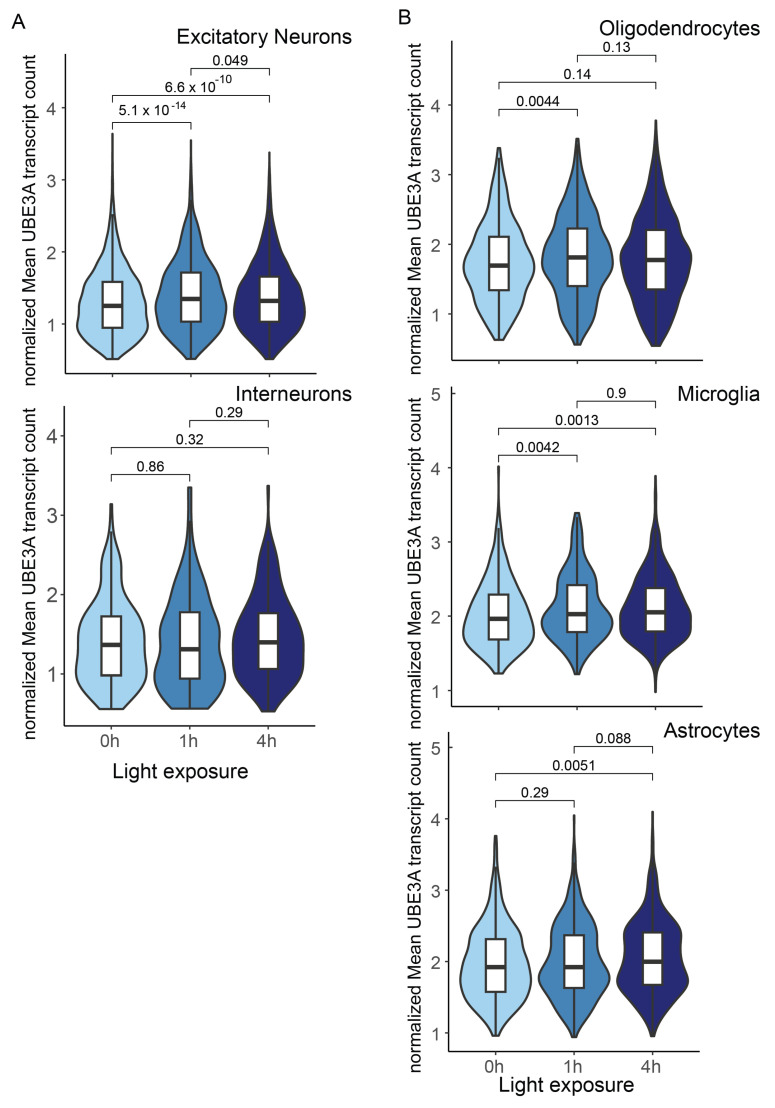
The visual stimulation induced *Ube3a* expression in the visual cortical area. The raw cell-by-gene matrix was downloaded from GSE102827. Both cell-type annotation and visual stimulus timing were used as indicated by the original authors. The *x*-axis represents the visual stimulus time, and the *y*-axis indicates the normalized UBE3A expression level. *T*-tests were performed to compare the different stimulus groups. (**A**) UBE3A expression in the excitatory neurons and interneurons. (**B**) UBE3A expression in glia: oligodendrocytes, microglia, and astrocytes.

Proper neuronal activity is crucial for brain development and cognitive functions. Neuronal activity either induces or suppresses the expression of the specific gene sets necessary for brain growth and function. Notably, UBE3A is such an activity-regulated gene. In vitro studies show that depolarization of primary neurons, stimulation by neurotrophins, and excitatory neurotransmitters can significantly induce the Myc-dependent expression of UBE3A [35]. Activity induced-UBE3A expression is crucial for kinase activation underlying learning and memory [36]. Interestingly, UBE3A upregulation is more pronounced in the nucleus, where the nuclear form of UBE3A is thought to be the primary factor contributing to Angelman syndrome [31]. It is suggested that UBE3A is also a coactivator of nuclear hormone receptors [37], which transcriptional regulates and induces the nuclei localization of nucleus hormone receptors. Strong evidence highlights the important roles of nuclear hormones and receptors in regulating normal brain development [38], myelination, and cognitive functions [39,40]. Notably, the UBE3A overexpression mouse models with extra copies of UBE3A exhibit distinct gene sets of upregulation that may be related to nuclear hormone receptors, genes on the X chromosome, and transcription factors that are sex-differentially regulated [41], particularly in glial cells such as astrocytes and oligodendrocytes. This could explain the observed sex-biased trend in autism [41]. Additionally, activity-regulated UBE3A expression has been observed in vivo. UBE3A is required for proper synaptic spine development [42,43,44] and experience-dependent maturation [43]. Visual stimulation caused a rapid increase in UBE3A expression across various cell types in the visual cortex [45], including excitatory neurons (Figure 3A) and glia cells, especially oligodendrocytes and microglia (Figure 3B), and astrocytes which increase later (Figure 3B). In the retina, where UBE3A is biallelically expressed in ganglion cells, higher levels of both paternal and maternal UBE3A in whole-cell lysates were observed. This increase was confirmed by immunofluorescent staining following exposure to white or blue light [46]. Therefore, it is reasonable to expect that UBE3A expression in the glia is regulated by neuronal activity during brain development, homeostatic regulation, and cognitive processes.

## 4. The Unconventional Secretory Form of UBE3A

Cerebrospinal fluid (CSF) biomarkers are frequently used in neurodegenerative and inflammatory diseases. Recently, molecular markers from CSF have been validated to detect, diagnose, and even predict therapeutic potential in neurodevelopmental disorders, including Angelman syndrome [47]. The catalytically active UBE3A protein was found in the CSF of wild-type rats and neurotypical human samples [48]. This suggests that cellular communication may depend on the secretion of UBE3A. When the UBE3A protein was supplied externally, it improved synaptic plasticity in brain sections of rats with Angelman syndrome and enhanced fear memory [49]. It was proposed that multiple isoforms of UBE3A exist in CSF, based on detecting the peptide sequences from different regions of the UBE3A protein [48]. The process by which UBE3A is secreted from donor cells and taken up by recipient cells remains unclear. It is also unknown whether neurons, glial cells, or both act as donors and receivers. Additionally, the specific subcellular location and the secretory pathway involved in UBE3A secretion are poorly understood. However, a promising area of exploration would be investigating the cellular communication—between neurons and neurons/glial cells—which is possibly mediated by the secretion and uptake of the UBE3A protein.

It was found that the catalytically active UBE3A in the extracellular space of the hippocampus could be dynamically regulated by neuronal activity [48]. Fear conditioning challenges or novel environmental stimulation significantly increased UBE3A expression and secretion. This raises an intriguing question of whether UBE3A specifically targets substrates in the extracellular matrix. Although the specific components remain unclear, a previous study identified a dramatic increase in the staining of perineuronal nets (PNN) using Wisteria floribunda agglutinin (WFA) in the dentate gyrus of flurothyl-kindled mice with Angelman syndrome [26]. The condition that causes seizures may be alleviated by the UBE3A reinstatement [26]. Some PNN proteins may be substrates of UBE3A that accumulate in the brains of individuals with Angelman syndrome. This accumulation may suppress inhibitory neurotransmission, leading to the induction of seizures. In Alzheimer’s disease, amyloid plaque is one of the typical pathological features that may be tightly regulated by UBE3A (discussed later). 

## 5. The Roles of UBE3A During Neurodevelopment

Many studies highlight the significance of UBE3A in brain development (see reviews [1,2,7,49,50]); here, we briefly emphasize the two roles of UBE3A. As an E3 ligase, UBE3A ubiquitinates multiple substrates, including Small Conductance Potassium Channel (SK2) [50], X-linked inhibitor of apoptosis (XIAP) [51], Ephexin5 [52], Brain and Muscle ARNT-Like 1 (BMAL1) [53], phosphotyrosyl phosphatase activator (PTPA) [54], and calcium- and voltage-dependent big potassium (BKα) channels [55], for ubiquitin-proteasome degradation. Notably, UBE3A regulates the UPS machinery through ubiquitinating PMSD4 (also known as Rpn10/S5a), with loss of UBE3A activity leading to decreased proteasome function [56]. Interestingly, UBE3A ubiquitination may not only be involved in protein degradation but also activity control, such as ALDH1A2 [57] and DDI1 [58]. Moreover, UBE3A regulates gene transcription, including the nuclear hormone receptor superfamily [37]. Such directions will be worthwhile to investigate further.

## 6. The Roles of UBE3A in Neurodegeneration

**Figure 4:**
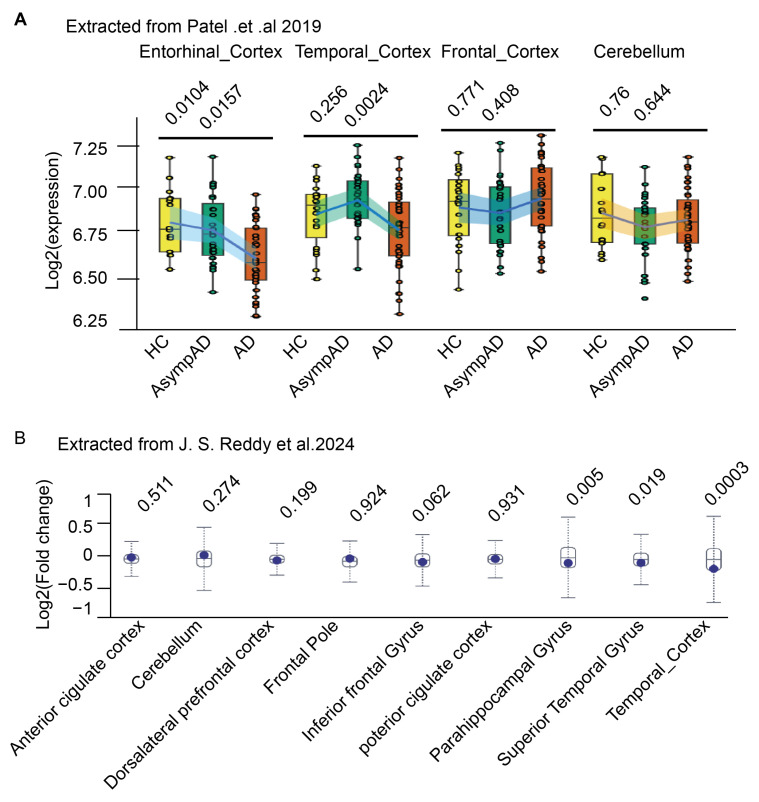
UBE3A expression in AD patients. Extracted RNA-seq data from the published literature were summarized and graphed. (**A**) Log2 expressions of UBE3A in healthy individuals, asymptomatic AD, and AD are compared by brain region [61]; (**B**) Log2-fold changes of UBE3A expression in healthy individuals and AD are compared by brain region [64]. The adjusted *p* value was shown on top of each group.

Alzheimer’s disease is the most common form of dementia, with a progressive loss of memory and cognitive function due to neurodegeneration, hyperphosphorylated Tau tangles, and amyloid plaques. Amyloid plaques are aggregated amyloid-β (Aβ) peptides, with multiple steps of procession from amyloid precursor protein (APP) [59]. APP regulates proper neuronal development [60]. The oligomers of Aβ downregulate UBE3A in primary cultures [21,22]. Transcriptomic studies [61,62,63,64] comparing healthy controls and AD patient brains showed significant decreases in UBE3A levels in multiple brain regions, including the entorhinal cortex (Figure 4A), temporal lobe, and hippocampal gyrus (Figure 4B). Cell type-specific analysis [63] revealed a more profound decrease in excitatory neurons. In the Tg2576 and APPswe/PS1 AD mouse models, soluble UBE3A decreased before cognitive impairments were observed [21,22]. Intriguingly, insoluble UBE3A was increased where insoluble Aβ was detected [22]. This implied UBE3A may play a role in mediating aggregation or Aβ degradation, as the Aβ plaque was reduced in UBE3A-deficient APPswe/PS1δE9 AD mice [22]. However, the loss of UBE3A exaggerated cognitive impairments in APPswe/PS1δE9 AD mice [22] and accounted for the decreased dendritic spine density and synaptic dysfunction in Tg2576 mice [21]. 

UBE3A may regulate the APP processing and secretion of Aβ peptide. In the animal model of Dup15q syndrome, where UBE3A is duplicated, the increased intraneuronal accumulation of N-terminally truncated Aβ peptide at endosomes, autophagic vacuoles, Lamp1-positive lysosomes, and lipofuscin has been observed by a microscopy study [65]. Recent evidence indicates that UBE4B [66], an E4 ubiquitin elongation enzyme, mitigates neurodegeneration in a Drosophila AD model by promoting the ubiquitination and autophagy-dependent degradation of Tau. Since hyperphosphorylated Tau speeds up Tau and Aβ aggregation, UBE3A may bridge autophagy and proteasome degradation [67]; further investigation of UBE3A in AD is worthwhile. 

**Figure 5:**
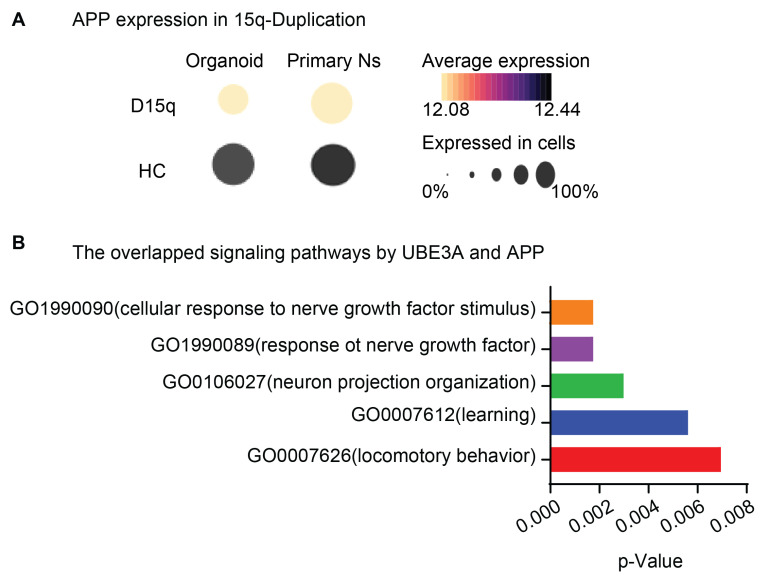
(**A**) Relative APP expression in Dup15q. The published literature [70] grouped relative APP from patients’ iPSC-derived organoids or postmortem primary cells by RNA-seq analysis. (**B**) Predicated signaling pathways that UBE3A and APP may share in neuronal functions. (https://www.ndexbio.org/iquery/, accessed at 17 Feb 2025).

UBE3A may directly regulate APP, in addition to its processing. It was found that Angelman syndrome patients (lacking neuronal UBE3A) have increased plasma APP and Aβ peptides compared to controls [23]. Autism patients are predicted to be over two times more likely to develop early-onset AD [68]. Interestingly, the modeling of Dup15q syndrome in vitro showed dramatically decreased APP levels [69]. Extracted data from a recent single-nuclei RNA-seq study [70] using brain organoids and frozen post-mortem tissues from dup15q patients and neurotypical controls also demonstrated an evident reduction in APP expression (Figure 5A). Importantly, UBE3A is one of the most abundant UPS-linked proteins that was observed in a proteomic study which was pulled down by the StrepTactin resin-linked APP cytosolic region [71]. Such experimental evidence demands further studies that directly investigate the interactions of UBE3A and APP. Together with the downregulation of UBE3A by Aβ oligomers, we propose that the potential regulation of APP by UBE3A reduces the Aβ production, which is dampened in the AD condition and further deteriorated by the accumulation of Aβ. 

UBE3A and APP may share signaling pathways crucial for brain functions. It is predicted (https://www.ndexbio.org/iquery/, accessed at 17 Feb 2025) that both UBE3A and APP are involved in pathways regulating neuronal/cellular responses to nerve growth factors, neural projection organization, learning, and locomotor skills (Figure 5B), all of which are frequently dysregulated in neurodevelopmental and neurodegenerative diseases. In addition, catalytically inactive forms of UBE3A may impair overall proteasome function due to the accumulation of the S5a proteasomal subunit [56]. Ubiquitin and proteasome dysfunctions are frequently reported in neurodegeneration [72]. In 3D human neural cell culture, ubiquitin signaling alternation accumulates APP and mimics AD pathology [73]. Importantly, enhancing proteasome functions has shown promise in preventing cognitive anomalies in the rTg4510 mouse model of progressive tauopathy [74]. It is worthwhile to further explore the involvement of UBE3A in such impairments and its potential to be druggable in AD and other neurodegenerations.

UBE3A may also play roles in other types of neurodegenerations. Abnormal dopamine signaling was found in an Angelman syndrome mouse model [75], where the loss of dopaminergic neurons disrupted the circuitry of the basal ganglia which contributed to motor dysfunction [76,77] and reward-seeking enhancement [78]. Disruption of dopamine signaling is typically observed in Parkinson’s disease. Investigating the UBE3A and the Parkin (Parkinson’s disease gene) simultaneously would benefit both diseases. 

Huntingtin (Htt)-associated protein 1 (HAP1) is implicated in Huntington’s disease (HD), which is caused by an increase in the CAG trinucleotide repeat expansion in the huntingtin gene (Htt) due to an autosomal dominant mutation on chromosome 4 (4p16.3). HAP1 accumulation increases autophagy influx in Angelman syndrome mice, and HAP1 is one of the UBE3A substrates [79]. Interestingly, UBE3A is hyposufficient in HD mice brains with globally increased aggregation, which has less ubiquitination of Htt [18]. K48-mediated ubiquitination and degradation of HTT fragments depends on UBE3A [19]. The viral expression of UBE3A in HD brains normalized the aggregation of mHtt protein and reduced the mHtt aggregation-induced cell death [19], which is blocked by UPS inhibitor MG132 [19]. Similarly, pharmacological interventions of HD mice by Azadiradione [80] and Topotecan [81] are correlated with UBE3A expression. Removal of UBE3A disrupts the Azadiradione-induced BDNF expression [82]. Topotecan is the first class of the identified small molecule silencers of paternal UBE3A [11]. Collectively, UBE3A manipulation may benefit HD therapeutics. Supporting this, removing UBE3A in HD mice accelerated disease pathology and caused motor phenotypes with a shorter lifespan [18]. 

Loss of UBE3A also exaggerates Purkinje cell loss in spinocerebellar ataxia type 1 (SCA1) mice [83]. Removing UBE3A enhances the polyglutamine protein aggregation and cell death [84]. The over-expression of UBE3A diminishes polyglutamine protein aggregation-induced cell death [84]. Similarly, the overexpression of UBE3A increases the ubiquitination and facilitates the degradation of superoxide dismutase 1 (SOD1) proteins, which is linked to familial amyotrophic lateral sclerosis (ALS) [85]. Moreover, stress-induced separation and proteasome foci were tightly correlated with UBE3A expression [86], in which overexpression protects misfolded protein-induced cell death [84]. 

In summary, UBE3A is positively involved in multiple neurodegenerative diseases where UBE3A normalization protects neuronal death. The co-localization of UBE3A with Htt aggregates [18,19,84], stress granules [86], SOD1 [85], Aβ plaques [22], and misfolded proteins [84] highlights the possibilities of targeting UBE3A for therapeutics. The proteolysis-targeting chimera (PROTAC) molecule, since the concept was proposed two decades ago [87], has revolutionized drug development, especially for undruggable diseases. The small molecule degrader harnesses the ubiquitin–proteasome system (alternatively, autophagy) [88] to degrade a target protein. Through a linker, PROTAC tags the target protein to an E3 ligase (alternatively, to an autophagosome, lysosome, or antibodies) [88]. Neurodegeneration, including tauopathy, HD [88], and AD [89] are under investigation with PROTAC. We believe that future studies designing UBE3A-specific PROTAC to target neurodegenerative diseases are worthwhile. 

## 7. The Glial Function of UBE3A: Neuronal Spine Morphology, Seizures, and More

There is no direct evidence indicating a loss of function of UBE3A in glial cells; however, there is increasing evidence of gain of function and gene overdosage effects, particularly in models with additional copies of maternal UBE3A. The UBE3A gene is located on human chromosome 15q11–13 and is highly susceptible to maternal duplication (dup15q) and triplication. This genetic variation is responsible for 1–3% of the autism population [90,91,92,93,94]. Mouse models with one or two extra copies of the UBE3A transgene, mimicking Dup15q syndrome, have shown key autism-like behaviors and decreased glutamatergic signaling [95]. Despite the overexpression of UBE3A in the nuclei, AMPA receptors (AMPARs) were not largely suppressed, yet associative learning and memory generally remained normal [96,97]. In contrast, significant seizure symptoms and difficulties in social interactions have been consistently reported [96,97,98,99]. Nuclear increases in UBE3A in neurons impair sociability by repressing Cbln1 gene expression, a key node in an autism-related gene network due to protein–protein interactions [98]. It remains uncertain whether such social abnormalities relate to glia. However, these deficits could be reversed by preclinical AAV viral vector gene therapy [98] or improved by crossing with the Angelman syndrome mouse model [97]. It was observed that UBE3A staining is lower in the oligodendroglia from a mouse model with a gain of function mutation linked to autism [100]. However, the effects of UBE3A in other glial cells, such as astrocytes, remain unknown. Additionally, UBE3A gain of function mutations are thought to be hyper-catalytic [16,93] and may even lead to the degradation of UBE3A itself [101]. There is a pressing need for mouse models that exhibit UBE3A overexpression and/or copy number variations in specific types of glial cells.

Neuronal morphological abnormalities, including reductions in the complexity of dendritic arborization and/or the density of dendritic spines, underlie neurodevelopment disorders and could potentially be druggable [42,102]. UBE3A-dependent spine morphology has often been reported in individuals with Angelman syndrome [42,44,102,103] and UBE3A overexpression [51]. Interestingly, primary rat neurons transfected with the UBE3A plasmid showed no morphological differences, although the exact amount of UBE3A remained unknown [104]. Primary astrocytes expressing UBE3A could increase immature dendritic protrusions in cocultured neurons while reducing spine density, which occurs when UBE3A is also overexpressed in the neurons of the coculture [104]. Such research indicates that the development of spines is differently regulated by neuronal and glial UBE3A. In neurons, UBE3A may be involved in both the creation and maturation of spines, while in astrocytes, UBE3A appears to have a more significant role in spine maturation. There is cross-talk between neurons and astrocytes that influences the development of neuronal spines, driven by UBE3A in astrocytes. Furthermore, the overexpression of UBE3A in neonates through the intraventricular injection of an astrocyte-specific virus is found to replicate spine morphology and induce autistic-like behaviors in mice [104]. Thrombospondins secreted by astrocytes play a crucial role in regulating synapse formation. It has been found that UBE3A controls the transcription of spinogenic factors such as thrombospondin-2 (TSP2). When UBE3A is present in excess in astrocytes, the amount of produced and secreted TSP2 is significantly reduced [104]. It is well documented that both developing and mature astrocytes play a crucial role in the formation, maturation, and refinement of synapses [105]. Co-culturing neurons with astrocytes or using astrocyte-conditioned media enhances synaptic formation and maturation. Conversely, diseases associated with astrocytes often lead to an excessive elimination of synapses. It would be valuable to investigate whether neurotrophic factors change through UBE3A in astrocytes. Additionally, we should examine any alterations in vivo in the interactions between astrocytes and neurons, particularly concerning tripartite synapses, which represent another significant way in which astrocytes directly regulate synapses [106]. 

Experience-driven dendritic spine maintenance was reported to be impaired in the Angelman syndrome mouse model [107]. Additionally, it was proposed that pre-synaptic UBE3A in Drosophila could eliminate synapse cells autonomously [108]. Mechanistically, the process by which developing synapses are removed remains unclear. If UBE3A functions are conserved from flies to mammals, the impaired maintenance of synapses observed in rodents is likely not solely a result of neuronal factors; rather, it may be influenced by glial cells. Astrocytes, microglia, and oligodendrocyte precursor cells can all engulf synapses to some extent [109]. However, the role of UBE3A in glial cells has been largely overlooked for decades. UBE3A interacts with interferon regulatory factor (IRF) and enhances IRF-dependent transcription in neurons [110]. The enrichment of genes downstream of IRF has been observed in a UBE3A-deficient mouse model of Angelman syndrome [110]. This finding suggests a novel function for UBE3A as a transcriptional regulator of the brain’s immune system. Future studies focusing on the role of UBE3A in microglia, the primary immune cell type in the brain, are essential. It remains unclear whether the available tools to study UBE3A loss or gain of function effectively address potential dysfunction in microglia and other glial cells. The dynamic interactions between neurons and glia are crucial for normal brain development and function [109]. Therefore, it is important to investigate whether UBE3A expression in glia changes in response to fluctuations in neuronal UBE3A levels. Additionally, exploring the impact of neuron-specific UBE3A overdosage or insufficiency in glial cells could provide valuable insights for therapeutic strategies. With advancements such as single-cell and single-nucleus RNA sequencing, along with other omics technologies, there is an opportunity to uncover more novel functions of UBE3A and to update our understanding of the mechanisms underlying the disorders associated with UBE3A loss or gain of function. Research utilizing single-nucleus RNA sequencing compared individuals with dup15q to neurotypical controls, revealing an increased UBE3A expression in the neurons, microglia, astrocytes, and oligodendroglia, which triggered the activation of various gene networks [41,111]. In microglia, an inflammatory transcriptional network was identified; however, further investigation is needed to determine the extent to which UBE3A isoform(s) directly contributes to this network [111].

Intriguingly, the overexpression of glial UBE3A directly contributes to the seizure phenotype in Drosophila flies [112,113], potentially mirroring situations seen in Dup15q syndrome, where dysfunction in both neuronal and glial physiology has been documented [111]. It is essential to distinguish the specific contributions of glia and neurons to develop effective mechanistic interventions. In the Drosophila model of Dup15q syndrome, it was demonstrated that only the glial-driven expression of the UBE3A ortholog dube3a directly led to a bang-sensitive seizure phenotype [112,113]. Both glial and neuronal-driven dube3a expression resulted in noticeable motor phenotypes. High temperatures prompted spontaneous seizure-associated immobilization events with both glial and neuronal-driven dube3a expression, and mechanical shocks triggered convulsions [112,113]. These findings suggest that glial dube3a contributes directly to hyperexcitability in flies [112]. Notably, while the overexpression of UBE3A is toxic to Drosophila, resulting in a shortened lifespan [112], the same overexpression appears to have a neutral effect in rodents. It remains unclear whether the induction of seizures due to glial UBE3A overexpression in flies is also observed in rodents and humans [16,93,99,100], and how translational these findings may be. Our limited knowledge of UBE3A substrates [103,114], along with a lack of a comprehensive understanding of the transcriptional networks regulated by UBE3A [115,116,117], leaves the molecular mechanisms underlying seizures and epilepsy in individuals with Dup15q and Angelman syndrome largely unknown.

## 8. Conclusions: A Yin–Yang Model of UBE3A Functions in Neurons and Glia

**Figure 6:**
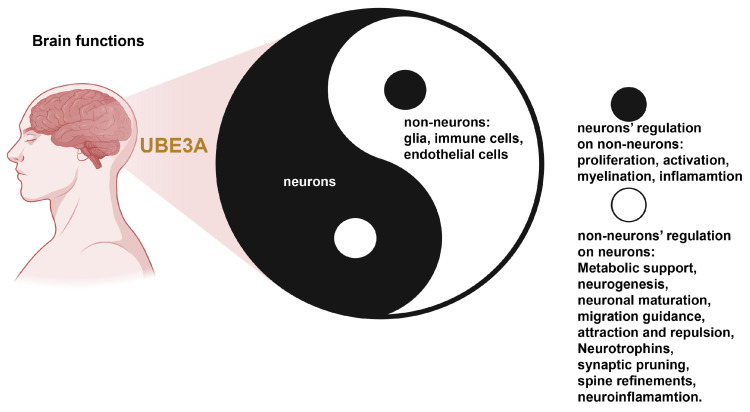
A Yin–Yang model of UBE3A functions in neurons and glia. This schematic illustrates the reciprocal regulation of neuronal (Yang) and glial (Yin) UBE3A, emphasizing their combined importance for normal brain function throughout development, aging, and in pathological states. Neuronal UBE3A is well known for its roles in synaptic pruning, plasticity, and maturation, but recent evidence suggests that glial UBE3A also contributes to these processes—potentially responding to secreted UBE3A from neurons, regulating glial proliferation, and influencing myelination. When balanced, these Yin–Yang interactions support healthy cognitive function; however, disruptions in either neuronal or glial UBE3A can destabilize the entire system, fueling the emergence of neurodevelopmental or neurodegenerative conditions.

Taken together, mounting evidence indicates that UBE3A maintains a delicate balance between neuronal and glial physiology. We propose a Yin–Yang framework (Figure 6) in which neuronal UBE3A (Yang) and glial UBE3A (Yin) reciprocally regulate each other, thereby sustaining optimal brain function across development, aging, and in pathological contexts.

In neurons, UBE3A can localize presynaptically to facilitate synaptic pruning [108], or within the nucleus to modulate hormone receptor transcription [37] and cell-cycle progression [118]. Its secretion by neurons [48] may further enhance synaptic plasticity [49], while potentially being taken up by glial cells such as astrocytes and oligodendroglia [104,112,113]. This cross-cell transfer could influence glial proliferation, differentiation, and apoptosis [119,120,121], and ultimately shape myelination in response to neuronal activity [122,123,124]. Despite these promising insights, the precise mechanisms by which UBE3A mediates neuron–glia interactions remain incompletely understood. An expanded focus on glial UBE3A—including cell-specific roles and uptake pathways—could uncover novel therapeutic targets for a range of brain disorders. 

Looking beyond the conventional neuron-centric view of UBE3A will be crucial for understanding how neuron–glia communication drives fundamental neural functions, including those essential for proper development and cognition. Although Angelman syndrome has provided a vivid illustration of how the loss of maternal UBE3A can derail these processes, the emerging recognition of UBE3A’s broader contributions across neurodevelopmental and neurodegenerative conditions calls for a deeper investigation of its glial and cross-cell activities. Crucially, because there are already multiple treatments for AS that aim to upregulate UBE3A, these strategies could be repurposed or adapted to address UBE3A deficits in other neurocognitive disorders such as Alzheimer’s disease and related neurodegenerative conditions. Collectively, these insights reinforce the vital role of UBE3A in synaptic and cognitive function, and underscore its promise for therapeutic intervention in a wide array of neurological conditions.

## Data Availability

No new data were generated for this article. Reanalysis of published datasets has been noted or cited in the references. Any additional data requests should be directed to the corresponding authors of the original studies.

## References

[1] Khago D., Fucci I.J., Byrd R.A. (2020). The Role of Conformational Dynamics in the Recognition and Regulation of Ubiquitination. Molecules.

[2] Barroso-Gomila O., Merino-Cacho L., Muratore V., Perez C., Taibi V., Maspero E., Azkargorta M., Iloro I., Trulsson F., Vertegaal A.C.O. (2023). BioE3 identifies specific substrates of ubiquitin E3 ligases. Nat. Commun..

[3] Chaudhary P., Proulx J., Park I.W. (2023). Ubiquitin-protein ligase E3A (UBE3A) mediation of viral infection and human diseases. Virus Res..

[4] Owais A., Mishra R.K., Kiyokawa H. (2020). The HECT E3 Ligase E6AP/UBE3A as a Therapeutic Target in Cancer and Neurological Disorders. Cancers.

[5] Samanta D. (2021). Epilepsy in Angelman syndrome: A scoping review. Brain Dev..

[6] Chamberlain S.J., Brannan C.I. (2001). The Prader-Willi syndrome imprinting center activates the paternally expressed murine Ube3a antisense transcript but represses paternal Ube3a. Genomics.

[7] Rougeulle C., Glatt H., Lalande M. (1997). The Angelman syndrome candidate gene, UBE3A/E6-AP, is imprinted in brain. Nat. Genet..

[8] Judson M.C., Sosa-Pagan J.O., Del Cid W.A., Han J.E., Philpot B.D. (2014). Allelic specificity of Ube3a expression in the mouse brain during postnatal development. J. Comp. Neurol..

[9] Dindot S.V., Christian S., Murphy W.J., Berent A., Panagoulias J., Schlafer A., Ballard J., Radeva K., Robinson R., Myers L. (2023). An ASO therapy for Angelman syndrome that targets an evolutionarily conserved region at the start of the UBE3A-AS transcript. Sci. Transl. Med..

[10] Meng L., Ward A.J., Chun S., Bennett C.F., Beaudet A.L., Rigo F. (2015). Towards a therapy for Angelman syndrome by targeting a long non-coding RNA. Nature.

[11] Huang H.S., Allen J.A., Mabb A.M., King I.F., Miriyala J., Taylor-Blake B., Sciaky N., Dutton J.W., Lee H.M., Chen X. (2011). Topoisomerase inhibitors unsilence the dormant allele of Ube3a in neurons. Nature.

[12] Meng L., Person R.E., Huang W., Zhu P.J., Costa-Mattioli M., Beaudet A.L. (2013). Truncation of Ube3a-ATS unsilences paternal Ube3a and ameliorates behavioral defects in the Angelman syndrome mouse model. PLoS Genet..

[13] Vihma H., Li K., Welton-Arndt A., Smith A.L., Bettadapur K.R., Gilmore R.B., Gao E., Cotney J.L., Huang H.C., Collins J.L. (2024). Ube3a unsilencer for the potential treatment of Angelman syndrome. Nat. Commun..

[14] Offensperger F., Muller F., Jansen J., Hammler D., Gotz K.H., Marx A., Sirois C.L., Chamberlain S.J., Stengel F., Scheffner M. (2020). Identification of Small-Molecule Activators of the Ubiquitin Ligase E6AP/UBE3A and Angelman Syndrome-Derived E6AP/UBE3A Variants. Cell Chem. Biol..

[15] Huang B., Zhou L., Liu R., Wang L., Xue S., Shi Y., Jeong G.H., Jeong I.H., Li S., Yin J. (2022). Activation of E6AP/UBE3A-Mediated Protein Ubiquitination and Degradation Pathways by a Cyclic gamma-AA Peptide. J. Med. Chem..

[16] Weston K.P., Gao X., Zhao J., Kim K.S., Maloney S.E., Gotoff J., Parikh S., Leu Y.C., Wu K.P., Shinawi M. (2021). Identification of disease-linked hyperactivating mutations in UBE3A through large-scale functional variant analysis. Nat. Commun..

[17] Salminen I., Read S., Hurd P., Crespi B. (2019). Genetic variation of UBE3A is associated with schizotypy in a population of typical individuals. Psychiatry Res..

[18] Maheshwari M., Shekhar S., Singh B.K., Jamal I., Vatsa N., Kumar V., Sharma A., Jana N.R. (2014). Deficiency of Ube3a in Huntington’s disease mice brain increases aggregate load and accelerates disease pathology. Hum. Mol. Genet..

[19] Bhat K.P., Yan S., Wang C.E., Li S., Li X.J. (2014). Differential ubiquitination and degradation of huntingtin fragments modulated by ubiquitin-protein ligase E3A. Proc. Natl. Acad. Sci. USA.

[20] Gu X., Hou Y., Chen Y., Ou R., Cao B., Wei Q., Zhang L., Song W., Zhao B., Wu Y. (2022). Enrichment of rare variants in E3 ubiquitin ligase genes in Early onset Parkinson’s disease. Neurobiol. Aging.

[21] Olabarria M., Pasini S., Corona C., Robador P., Song C., Patel H., Lefort R. (2019). Dysfunction of the ubiquitin ligase E3A Ube3A/E6-AP contributes to synaptic pathology in Alzheimer’s disease. Commun. Biol..

[22] Singh B.K., Vatsa N., Kumar V., Shekhar S., Sharma A., Jana N.R. (2017). Ube3a deficiency inhibits amyloid plaque formation in APPswe/PS1deltaE9 mouse model of Alzheimer’s disease. Hum. Mol. Genet..

[23] Erickson C.A., Wink L.K., Baindu B., Ray B., Schaefer T.L., Pedapati E.V., Lahiri D.K. (2016). Analysis of peripheral amyloid precursor protein in Angelman Syndrome. Am. J. Med. Genet. A.

[24] Sonzogni M., Zhai P., Mientjes E.J., van Woerden G.M., Elgersma Y. (2020). Assessing the requirements of prenatal UBE3A expression for rescue of behavioral phenotypes in a mouse model for Angelman syndrome. Mol. Autism.

[25] Silva-Santos S., van Woerden G.M., Bruinsma C.F., Mientjes E., Jolfaei M.A., Distel B., Kushner S.A., Elgersma Y. (2015). Ube3a reinstatement identifies distinct developmental windows in a murine Angelman syndrome model. J. Clin. Investig..

[26] Gu B., Carstens K.E., Judson M.C., Dalton K.A., Rougie M., Clark E.P., Dudek S.M., Philpot B.D. (2019). Ube3a reinstatement mitigates epileptogenesis in Angelman syndrome model mice. J. Clin. Investig..

[27] Wolter J.M., Mao H., Fragola G., Simon J.M., Krantz J.L., Bazick H.O., Oztemiz B., Stein J.L., Zylka M.J. (2020). Cas9 gene therapy for Angelman syndrome traps Ube3a-ATS long non-coding RNA. Nature.

[28] Grier M.D., Carson R.P., Lagrange A.H. (2015). Toward a Broader View of Ube3a in a Mouse Model of Angelman Syndrome: Expression in Brain, Spinal Cord, Sciatic Nerve and Glial Cells. PLoS ONE.

[29] Burette A.C., Judson M.C., Li A.N., Chang E.F., Seeley W.W., Philpot B.D., Weinberg R.J. (2018). Subcellular organization of UBE3A in human cerebral cortex. Mol. Autism.

[30] Williams K., Irwin D.A., Jones D.G., Murphy K.M. (2010). Dramatic Loss of Ube3A Expression during Aging of the Mammalian Cortex. Front. Aging Neurosci..

[31] Avagliano Trezza R., Sonzogni M., Bossuyt S.N.V., Zampeta F.I., Punt A.M., van den Berg M., Rotaru D.C., Koene L.M.C., Munshi S.T., Stedehouder J. (2019). Loss of nuclear UBE3A causes electrophysiological and behavioral deficits in mice and is associated with Angelman syndrome. Nat. Neurosci..

[32] Valluy J., Bicker S., Aksoy-Aksel A., Lackinger M., Sumer S., Fiore R., Wust T., Seffer D., Metge F., Dieterich C. (2015). A coding-independent function of an alternative Ube3a transcript during neuronal development. Nat. Neurosci..

[33] Zhang Y., Chen K., Sloan S.A., Bennett M.L., Scholze A.R., O’Keeffe S., Phatnani H.P., Guarnieri P., Caneda C., Ruderisch N. (2014). An RNA-sequencing transcriptome and splicing database of glia, neurons, and vascular cells of the cerebral cortex. J. Neurosci..

[34] Zhang Y., Sloan S.A., Clarke L.E., Caneda C., Plaza C.A., Blumenthal P.D., Vogel H., Steinberg G.K., Edwards M.S., Li G. (2016). Purification and Characterization of Progenitor and Mature Human Astrocytes Reveals Transcriptional and Functional Differences with Mouse. Neuron.

[35] Greer P.L., Hanayama R., Bloodgood B.L., Mardinly A.R., Lipton D.M., Flavell S.W., Kim T.K., Griffith E.C., Waldon Z., Maehr R. (2010). The Angelman Syndrome protein Ube3A regulates synapse development by ubiquitinating arc. Cell.

[36] Filonova I., Trotter J.H., Banko J.L., Weeber E.J. (2014). Activity-dependent changes in MAPK activation in the Angelman Syndrome mouse model. Learn. Mem..

[37] Nawaz Z., Lonard D.M., Smith C.L., Lev-Lehman E., Tsai S.Y., Tsai M.J., O’Malley B.W. (1999). The Angelman syndrome-associated protein, E6-AP, is a coactivator for the nuclear hormone receptor superfamily. Mol. Cell. Biol..

[38] Olivares A.M., Moreno-Ramos O.A., Haider N.B. (2015). Role of Nuclear Receptors in Central Nervous System Development and Associated Diseases. J. Exp. Neurosci..

[39] Chen J.F., Liu K., Hu B., Li R.R., Xin W., Chen H., Wang F., Chen L., Li R.X., Ren S.Y. (2021). Enhancing myelin renewal reverses cognitive dysfunction in a murine model of Alzheimer’s disease. Neuron.

[40] Ren S.Y., Xia Y., Yu B., Lei Q.J., Hou P.F., Guo S., Wu S.L., Liu W., Yang S.F., Jiang Y.B. (2024). Growth hormone promotes myelin repair after chronic hypoxia via triggering pericyte-dependent angiogenesis. Neuron.

[41] Montani C., Balasco L., Pagani M., Alvino F.G., Barsotti N., de Guzman A.E., Galbusera A., de Felice A., Nickl-Jockschat T.K., Migliarini S. (2024). Sex-biasing influence of autism-associated Ube3a gene overdosage at connectomic, behavioral, and transcriptomic levels. Sci. Adv..

[42] Yang X. (2020). Characterizing spine issues: If offers novel therapeutics to Angelman syndrome. Dev. Neurobiol..

[43] Yashiro K., Riday T.T., Condon K.H., Roberts A.C., Bernardo D.R., Prakash R., Weinberg R.J., Ehlers M.D., Philpot B.D. (2009). Ube3a is required for experience-dependent maturation of the neocortex. Nat. Neurosci..

[44] Dindot S.V., Antalffy B.A., Bhattacharjee M.B., Beaudet A.L. (2008). The Angelman syndrome ubiquitin ligase localizes to the synapse and nucleus, and maternal deficiency results in abnormal dendritic spine morphology. Hum. Mol. Genet..

[45] Hrvatin S., Hochbaum D.R., Nagy M.A., Cicconet M., Robertson K., Cheadle L., Zilionis R., Ratner A., Borges-Monroy R., Klein A.M. (2018). Single-cell analysis of experience-dependent transcriptomic states in the mouse visual cortex. Nat. Neurosci..

[46] Lin C.W., Cheng Y.C., Yang C.H., Huang H.S. (2023). Light activates Ube3a, an Angelman syndrome-associated gene, by mediating the chromatin structures during postnatal development of mouse retina. J. Neurochem..

[47] Hawtin R., Couthouis J., Khan S., Wang G., Panagoulias J., Gold L., Daugherty S., Kunecki D., Berry-Kravis E., Berent A. (2024). Proteomic Profiling of Angelman Syndrome for Disease-associated Biomarker Discovery (P8-8.001). Neurology.

[48] Dodge A., Willman J., Willman M., Nenninger A.W., Morrill N.K., Lamens K., Greene H., Weeber E.J., Nash K.R. (2021). Identification of UBE3A Protein in CSF and Extracellular Space of the Hippocampus Suggest a Potential Novel Function in Synaptic Plasticity. Autism Res..

[49] Dodge A., Morrill N.K., Weeber E.J., Nash K.R. (2022). Recovery of Angelman syndrome rat deficits with UBE3A protein supplementation. Mol. Cell. Neurosci..

[50] Sun J., Zhu G., Liu Y., Standley S., Ji A., Tunuguntla R., Wang Y., Claus C., Luo Y., Baudry M. (2015). UBE3A Regulates Synaptic Plasticity and Learning and Memory by Controlling SK2 Channel Endocytosis. Cell Rep..

[51] Khatri N., Gilbert J.P., Huo Y., Sharaflari R., Nee M., Qiao H., Man H.Y. (2018). The Autism Protein Ube3A/E6AP Remodels Neuronal Dendritic Arborization via Caspase-Dependent Microtubule Destabilization. J. Neurosci..

[52] Margolis S.S., Salogiannis J., Lipton D.M., Mandel-Brehm C., Wills Z.P., Mardinly A.R., Hu L., Greer P.L., Bikoff J.B., Ho H.Y. (2010). EphB-mediated degradation of the RhoA GEF Ephexin5 relieves a developmental brake on excitatory synapse formation. Cell.

[53] Gossan N.C., Zhang F., Guo B., Jin D., Yoshitane H., Yao A., Glossop N., Zhang Y.Q., Fukada Y., Meng Q.J. (2014). The E3 ubiquitin ligase UBE3A is an integral component of the molecular circadian clock through regulating the BMAL1 transcription factor. Nucleic Acids Res..

[54] Wang J., Lou S.S., Wang T., Wu R.J., Li G., Zhao M., Lu B., Li Y.Y., Zhang J., Cheng X. (2019). UBE3A-mediated PTPA ubiquitination and degradation regulate PP2A activity and dendritic spine morphology. Proc. Natl. Acad. Sci. USA.

[55] Sun A.X., Yuan Q., Fukuda M., Yu W., Yan H., Lim G.G.Y., Nai M.H., D’Agostino G.A., Tran H.D., Itahana Y. (2019). Potassium channel dysfunction in human neuronal models of Angelman syndrome. Science.

[56] Tomaic V., Banks L. (2015). Angelman syndrome-associated ubiquitin ligase UBE3A/E6AP mutants interfere with the proteolytic activity of the proteasome. Cell Death Dis..

[57] Xu X., Li C., Gao X., Xia K., Guo H., Li Y., Hao Z., Zhang L., Gao D., Xu C. (2018). Excessive UBE3A dosage impairs retinoic acid signaling and synaptic plasticity in autism spectrum disorders. Cell Res..

[58] Elu N., Osinalde N., Beaskoetxea J., Ramirez J., Lectez B., Aloria K., Rodriguez J.A., Arizmendi J.M., Mayor U. (2019). Detailed Dissection of UBE3A-Mediated DDI1 Ubiquitination. Front. Physiol..

[59] O’Brien R.J., Wong P.C. (2011). Amyloid precursor protein processing and Alzheimer’s disease. Annu. Rev. Neurosci..

[60] Muller U.C., Deller T., Korte M. (2017). Not just amyloid: Physiological functions of the amyloid precursor protein family. Nat. Rev. Neurosci..

[61] Patel H., Hodges A.K., Curtis C., Lee S.H., Troakes C., Dobson R.J.B., Newhouse S.J. (2019). Transcriptomic analysis of probable asymptomatic and symptomatic alzheimer brains. Brain Behav. Immun..

[62] Ghose U., Sproviero W., Winchester L., Amin N., Zhu T., Newby D., Ulm B.S., Papathanasiou A., Shi L., Liu Q. (2025). Genome wide association neural networks (GWANN) identify genes linked to family history of Alzheimer’s disease. Brief. Bioinform..

[63] Li X.W., Duan T.T., Chu J.Y., Pan S.Y., Zeng Y., Hu F.F. (2023). SCAD-Brain: A public database of single cell RNA-seq data in human and mouse brains with Alzheimer’s disease. Front. Aging Neurosci..

[64] Reddy J.S., Heath L., Linden A.V., Allen M., Lopes K.P., Seifar F., Wang E., Ma Y., Poehlman W.L., Quicksall Z.S. (2024). Bridging the gap: Multi-omics profiling of brain tissue in Alzheimer’s disease and older controls in multi-ethnic populations. Alzheimers Dement..

[65] Wegiel J., Frackowiak J., Mazur-Kolecka B., Schanen N.C., Cook E.H., Sigman M., Brown W.T., Kuchna I., Wegiel J., Nowicki K. (2012). Abnormal intracellular accumulation and extracellular Abeta deposition in idiopathic and Dup15q11.2-q13 autism spectrum disorders. PLoS ONE.

[66] Subramanian M., Hyeon S.J., Das T., Suh Y.S., Kim Y.K., Lee J.S., Song E.J., Ryu H., Yu K. (2021). UBE4B, a microRNA-9 target gene, promotes autophagy-mediated Tau degradation. Nat. Commun..

[67] Yang X., Duckhorn J., Marshall J., Huang Y.A. (2024). Interlinked destinies: How ubiquitin-proteasome and autophagy systems underpin neurocognitive outcomes. Exp. Neurol..

[68] Vivanti G., Tao S., Lyall K., Robins D.L., Shea L.L. (2021). The prevalence and incidence of early-onset dementia among adults with autism spectrum disorder. Autism Res..

[69] Baron C.A., Tepper C.G., Liu S.Y., Davis R.R., Wang N.J., Schanen N.C., Gregg J.P. (2006). Genomic and functional profiling of duplicated chromosome 15 cell lines reveal regulatory alterations in UBE3A-associated ubiquitin-proteasome pathway processes. Hum. Mol. Genet..

[70] Perez Y., Velmeshev D., Wang L., White M., Siebert C., Baltazar J., Dutton N.G., Wang S., Haeussler M., Chamberlain S. (2023). Single cell analysis of dup15q syndrome reveals developmental and postnatal molecular changes in autism. bioRxiv.

[71] Del Prete D., Rice R.C., Rajadhyaksha A.M., D’Adamio L. (2016). Amyloid Precursor Protein (APP) May Act as a Substrate and a Recognition Unit for CRL4CRBN and Stub1 E3 Ligases Facilitating Ubiquitination of Proteins Involved in Presynaptic Functions and Neurodegeneration. J. Biol. Chem..

[72] Schmidt M.F., Gan Z.Y., Komander D., Dewson G. (2021). Ubiquitin signalling in neurodegeneration: Mechanisms and therapeutic opportunities. Cell Death Differ..

[73] Maniv I., Sarji M., Bdarneh A., Feldman A., Ankawa R., Koren E., Magid-Gold I., Reis N., Soteriou D., Salomon-Zimri S. (2023). Altered ubiquitin signaling induces Alzheimer’s disease-like hallmarks in a three-dimensional human neural cell culture model. Nat. Commun..

[74] Myeku N., Clelland C.L., Emrani S., Kukushkin N.V., Yu W.H., Goldberg A.L., Duff K.E. (2016). Tau-driven 26S proteasome impairment and cognitive dysfunction can be prevented early in disease by activating cAMP-PKA signaling. Nat. Med..

[75] Riday T.T., Dankoski E.C., Krouse M.C., Fish E.W., Walsh P.L., Han J.E., Hodge C.W., Wightman R.M., Philpot B.D., Malanga C.J. (2012). Pathway-specific dopaminergic deficits in a mouse model of Angelman syndrome. J. Clin. Investig..

[76] Mulherkar S.A., Jana N.R. (2010). Loss of dopaminergic neurons and resulting behavioural deficits in mouse model of Angelman syndrome. Neurobiol. Dis..

[77] Hayrapetyan V., Castro S., Sukharnikova T., Yu C., Cao X., Jiang Y.H., Yin H.H. (2014). Region-specific impairments in striatal synaptic transmission and impaired instrumental learning in a mouse model of Angelman syndrome. Eur. J. Neurosci..

[78] Berrios J., Stamatakis A.M., Kantak P.A., McElligott Z.A., Judson M.C., Aita M., Rougie M., Stuber G.D., Philpot B.D. (2016). Loss of UBE3A from TH-expressing neurons suppresses GABA co-release and enhances VTA-NAc optical self-stimulation. Nat. Commun..

[79] Wang T., Wang J., Wang J., Mao L., Tang B., Vanderklish P.W., Liao X., Xiong Z.Q., Liao L. (2019). HAP1 is an in vivo UBE3A target that augments autophagy in a mouse model of Angelman syndrome. Neurobiol. Dis..

[80] Singh B.K., Vatsa N., Nelson V.K., Kumar V., Kumar S.S., Mandal S.C., Pal M., Jana N.R. (2018). Azadiradione Restores Protein Quality Control and Ameliorates the Disease Pathogenesis in a Mouse Model of Huntington’s Disease. Mol. Neurobiol..

[81] Shekhar S., Vatsa N., Kumar V., Singh B.K., Jamal I., Sharma A., Jana N.R. (2017). Topoisomerase 1 inhibitor topotecan delays the disease progression in a mouse model of Huntington’s disease. Hum. Mol. Genet..

[82] Jana S., Giri B., Das S., Manna A., Mandal S.C., Ranjan Jana N. (2024). Azadiradione up-regulates the expression of parvalbumin and BDNF via Ube3a. Gene.

[83] Cummings C.J., Reinstein E., Sun Y., Antalffy B., Jiang Y., Ciechanover A., Orr H.T., Beaudet A.L., Zoghbi H.Y. (1999). Mutation of the E6-AP ubiquitin ligase reduces nuclear inclusion frequency while accelerating polyglutamine-induced pathology in SCA1 mice. Neuron.

[84] Mishra A., Dikshit P., Purkayastha S., Sharma J., Nukina N., Jana N.R. (2008). E6-AP promotes misfolded polyglutamine proteins for proteasomal degradation and suppresses polyglutamine protein aggregation and toxicity. J. Biol. Chem..

[85] Mishra A., Maheshwari M., Chhangani D., Fujimori-Tonou N., Endo F., Joshi A.P., Jana N.R., Yamanaka K. (2013). E6-AP association promotes SOD1 aggresomes degradation and suppresses toxicity. Neurobiol. Aging.

[86] Yasuda S., Tsuchiya H., Kaiho A., Guo Q., Ikeuchi K., Endo A., Arai N., Ohtake F., Murata S., Inada T. (2020). Stress- and ubiquitylation-dependent phase separation of the proteasome. Nature.

[87] Sakamoto K.M., Kim K.B., Kumagai A., Mercurio F., Crews C.M., Deshaies R.J. (2001). Protacs: Chimeric molecules that target proteins to the Skp1-Cullin-F box complex for ubiquitination and degradation. Proc. Natl. Acad. Sci. USA.

[88] Bekes M., Langley D.R., Crews C.M. (2022). PROTAC targeted protein degraders: The past is prologue. Nat. Rev. Drug Discov..

[89] Kurihara T., Asahi T., Sawamura N. (2020). Cereblon-mediated degradation of the amyloid precursor protein via the ubiquitin-proteasome pathway. Biochem. Biophys. Res. Commun..

[90] Krumm N., O’Roak B.J., Shendure J., Eichler E.E. (2014). A de novo convergence of autism genetics and molecular neuroscience. Trends Neurosci..

[91] Hogart A., Wu D., LaSalle J.M., Schanen N.C. (2010). The comorbidity of autism with the genomic disorders of chromosome 15q11.2-q13. Neurobiol. Dis..

[92] Glessner J.T., Wang K., Cai G., Korvatska O., Kim C.E., Wood S., Zhang H., Estes A., Brune C.W., Bradfield J.P. (2009). Autism genome-wide copy number variation reveals ubiquitin and neuronal genes. Nature.

[93] Yi J.J., Berrios J., Newbern J.M., Snider W.D., Philpot B.D., Hahn K.M., Zylka M.J. (2015). An Autism-Linked Mutation Disables Phosphorylation Control of UBE3A. Cell.

[94] De Rubeis S., He X., Goldberg A.P., Poultney C.S., Samocha K., Cicek A.E., Kou Y., Liu L., Fromer M., Walker S. (2014). Synaptic, transcriptional and chromatin genes disrupted in autism. Nature.

[95] Smith S.E., Zhou Y.D., Zhang G., Jin Z., Stoppel D.C., Anderson M.P. (2011). Increased gene dosage of Ube3a results in autism traits and decreased glutamate synaptic transmission in mice. Sci. Transl. Med..

[96] Tian Y., Yu F., Yun E., Lin J.W., Man H.Y. (2024). mRNA nuclear retention reduces AMPAR expression and promotes autistic behavior in UBE3A-overexpressing mice. EMBO Rep..

[97] Punt A.M., Judson M.C., Sidorov M.S., Williams B.N., Johnson N.S., Belder S., den Hertog D., Davis C.R., Feygin M.S., Lang P.F. (2022). Molecular and behavioral consequences of Ube3a gene overdosage in mice. JCI Insight.

[98] Krishnan V., Stoppel D.C., Nong Y., Johnson M.A., Nadler M.J., Ozkaynak E., Teng B.L., Nagakura I., Mohammad F., Silva M.A. (2017). Autism gene Ube3a and seizures impair sociability by repressing VTA Cbln1. Nature.

[99] Copping N.A., Christian S.G.B., Ritter D.J., Islam M.S., Buscher N., Zolkowska D., Pride M.C., Berg E.L., LaSalle J.M., Ellegood J. (2017). Neuronal overexpression of Ube3a isoform 2 causes behavioral impairments and neuroanatomical pathology relevant to 15q11.2-q13.3 duplication syndrome. Hum. Mol. Genet..

[100] Xing L., Simon J.M., Ptacek T.S., Yi J.J., Loo L., Mao H., Wolter J.M., McCoy E.S., Paranjape S.R., Taylor-Blake B. (2023). Autism-linked UBE3A gain-of-function mutation causes interneuron and behavioral phenotypes when inherited maternally or paternally in mice. Cell Rep..

[101] Nuber U., Schwarz S.E., Scheffner M. (1998). The ubiquitin-protein ligase E6-associated protein (E6-AP) serves as its own substrate. Eur. J. Biochem..

[102] Sell G.L., Xin W., Cook E.K., Zbinden M.A., Schaffer T.B., O’Meally R.N., Cole R.N., Margolis S.S. (2021). Deleting a UBE3A substrate rescues impaired hippocampal physiology and learning in Angelman syndrome mice. Sci. Rep..

[103] Yang X. (2020). Towards an understanding of Angelman syndrome in mice studies. J. Neurosci. Res..

[104] Gardner Z., Holbrook O., Tian Y., Odamah K., Man H.Y. (2024). The role of glia in the dysregulation of neuronal spinogenesis in Ube3a-dependent ASD. Exp. Neurol..

[105] Chung W.S., Allen N.J., Eroglu C. (2015). Astrocytes Control Synapse Formation, Function, and Elimination. Cold Spring Harb. Perspect. Biol..

[106] Perea G., Navarrete M., Araque A. (2009). Tripartite synapses: Astrocytes process and control synaptic information. Trends Neurosci..

[107] Kim H., Kunz P.A., Mooney R., Philpot B.D., Smith S.L. (2016). Maternal Loss of Ube3a Impairs Experience-Driven Dendritic Spine Maintenance in the Developing Visual Cortex. J. Neurosci..

[108] Furusawa K., Ishii K., Tsuji M., Tokumitsu N., Hasegawa E., Emoto K. (2023). Presynaptic Ube3a E3 ligase promotes synapse elimination through down-regulation of BMP signaling. Science.

[109] Liu Y., Shen X., Zhang Y., Zheng X., Cepeda C., Wang Y., Duan S., Tong X. (2023). Interactions of glial cells with neuronal synapses, from astrocytes to microglia and oligodendrocyte lineage cells. Glia.

[110] Furumai R., Tamada K., Liu X., Takumi T. (2019). UBE3A regulates the transcription of IRF, an antiviral immunity. Hum. Mol. Genet..

[111] Dias C., Mo A., Cai C., Sun L., Cabral K., Brownstein C.A., Rockowitz S., Walsh C.A. (2024). Cell-type-specific effects of autism-associated 15q duplication syndrome in the human brain. Am. J. Hum. Genet..

[112] Hope K.A., LeDoux M.S., Reiter L.T. (2017). Glial overexpression of Dube3a causes seizures and synaptic impairments in Drosophila concomitant with down regulation of the Na(+)/K(+) pump ATPalpha. Neurobiol. Dis..

[113] Landaverde S., Sleep M., Lacoste A., Tan S., Schuback R., Reiter L.T., Iyengar A. (2024). Glial expression of Drosophila UBE3A causes spontaneous seizures that can be modulated by 5-HT signaling. Neurobiol. Dis..

[114] Sell G.L., Margolis S.S. (2015). From UBE3A to Angelman syndrome: A substrate perspective. Front. Neurosci..

[115] Lopez S.J., Segal D.J., LaSalle J.M. (2018). UBE3A: An E3 Ubiquitin Ligase With Genome-Wide Impact in Neurodevelopmental Disease. Front. Mol. Neurosci..

[116] Lopez S.J., Laufer B.I., Beitnere U., Berg E.L., Silverman J.L., O’Geen H., Segal D.J., LaSalle J.M. (2019). Imprinting effects of UBE3A loss on synaptic gene networks and Wnt signaling pathways. Hum. Mol. Genet..

[117] Martinez-Noel G., Luck K., Kuhnle S., Desbuleux A., Szajner P., Galligan J.T., Rodriguez D., Zheng L., Boyland K., Leclere F. (2018). Network Analysis of UBE3A/E6AP-Associated Proteins Provides Connections to Several Distinct Cellular Processes. J. Mol. Biol..

[118] Singhmar P., Kumar A. (2011). Angelman syndrome protein UBE3A interacts with primary microcephaly protein ASPM, localizes to centrosomes and regulates chromosome segregation. PLoS ONE.

[119] Luo Y., Yang Y., Yang C., Li C., Hu R., Geng W., Kang X., Lin H. (2023). UBE3A and MCM6 synergistically regulate the proliferation and migration of lung adenocarcinoma cells. FEBS Open Bio.

[120] Estridge R.C., Yagci Z.B., Sen D., Ptacek T.S., Simon J.M., Keung A.J. (2024). Loss of *UBE3A* impacts both neuronal and non-neuronal cells in human cerebral organoids. bioRxiv.

[121] Simchi L., Gupta P.K., Feuermann Y., Kaphzan H. (2023). Elevated ROS levels during the early development of Angelman syndrome alter the apoptotic capacity of the developing neural precursor cells. Mol. Psychiatry.

[122] Gibson E.M., Purger D., Mount C.W., Goldstein A.K., Lin G.L., Wood L.S., Inema I., Miller S.E., Bieri G., Zuchero J.B. (2014). Neuronal activity promotes oligodendrogenesis and adaptive myelination in the mammalian brain. Science.

[123] Gautier H.O., Evans K.A., Volbracht K., James R., Sitnikov S., Lundgaard I., James F., Lao-Peregrin C., Reynolds R., Franklin R.J. (2015). Neuronal activity regulates remyelination via glutamate signalling to oligodendrocyte progenitors. Nat. Commun..

[124] Marisca R., Hoche T., Agirre E., Hoodless L.J., Barkey W., Auer F., Castelo-Branco G., Czopka T. (2020). Functionally distinct subgroups of oligodendrocyte precursor cells integrate neural activity and execute myelin formation. Nat. Neurosci..

